# Child opinions related to a core outcome set for school-based healthy lifestyle behavior interventions: the COCOS study

**DOI:** 10.3389/fpubh.2025.1519467

**Published:** 2025-04-16

**Authors:** Teatske M. Altenburg, Lotte W. de Vries, Kheana Barbeau, Alyssa Button, Ashley Cox, Louise de Lannoy, Mhairi MacDonald, Rowena Naidoo, Amanda E. Staiano, Mark S. Tremblay, Deirdre M. Harrington

**Affiliations:** ^1^Amsterdam UMC Location Vrije Universiteit Amsterdam, Public and Occupational Health, Amsterdam, Netherlands; ^2^Amsterdam Public Health, Health Behaviours and Chronic Disease, Methodology, Amsterdam, Netherlands; ^3^Werklund School of Education, University of Calgary, Calgary, AB, Canada; ^4^Pennington Biomedical Research Center, Louisiana State University, Baton Rouge, LA, United States; ^5^Division of Musculoskeletal and Dermatological Sciences, Faculty of Biology, Medicine and Health, The University of Manchester, Manchester, United Kingdom; ^6^Healthy Active Living and Obesity (HALO) Research Group, Children's Hospital of Eastern Ontario Research Institute, Ottawa, ON, Canada; ^7^Sport, Physical Activity, Health and Wellbeing Research Group, Department of Sport and Physical Activity, Edge Hill University, Ormskirk, United Kingdom; ^8^Exercise and Leisure Sciences, College of Health Sciences, University of KwaZulu-Natal, Durban, South Africa; ^9^Department of Psychological Sciences and Health, University of Strathclyde, Glasgow, United Kingdom; ^10^Diabetes Research Centre, University of Leicester, Leicester, United Kingdom

**Keywords:** obesity prevention, importance ratings, outcomes, children's perspectives, international

## Abstract

**Introduction:**

Including children's perspectives developing health programs is a priority. This study gathered children's perspectives on outcomes in a Core Outcomes Set (COS), which they believe are important to measure in school-based healthy lifestyle behavioral interventions.

**Methods:**

Children aged 8–12 years from six countries across three continents participated in standardized interactive focus groups. An animation video was used to explain all relevant concepts (e.g., “intervention”, “outcomes”) and showed animated children engaging in a variety of lifestyle behaviors at school. Participating children then brainstormed and proposed outcomes they consider important to measure when evaluating a school-based “healthy lifestyle programme”. Next, children individually rated the importance of the outcomes using a traffic light system (red, “not important”; orange, “important”; green, “very important”). Similar outcomes (across focus groups and countries) were merged, and an overall importance rating was given to each outcome (across countries and overall). An outcome was considered important for inclusion in a COS if ≥70% of children scored the outcome as “very important” and <15% scored it as “not important”.

**Results:**

Children (*n* = 159) proposed 170 unique outcomes. Children proposed thirty-six outcomes in at least two countries, of which 20 outcomes received an overall rating of “very important” in all countries where the outcomes were reported. Of these 20, five outcomes were reported by children in at least four countries: being healthy, healthy diet, concentration, having fun, and feeling happy.

**Conclusion:**

Children reported a wide range of outcomes related to physical and mental health, as well as enjoyment and social skills, such as having fun and making friends. All outcomes reported by children in at least two countries and considered “very important” will be considered for inclusion in the consensus stage of developing a COS for school-based intervention studies aimed at childhood overweight and obesity prevention.

## Introduction

Prevention of childhood overweight and obesity is an international public health priority (1) given that worldwide prevalence rates have increased in recent decades in high- and low-to-middle-income countries (2, 3). Numerous interventions have been developed and tested to prevent childhood overweight, mostly in school settings, because most children can be reached through schools, and schools provide an optimal setting for primary prevention implementation (4–6).

Meta-analyses of studies pooling the results of school-based overweight prevention interventions are important to summarize evidence on effective intervention strategies. However, such meta-analyses often exclude relevant studies because the required outcome, such as objectively measured height and weight using anthropometry, is not reported (7, 8). In a recent scoping review, we observed that a wide variety of outcomes, such as BMI, BMI-z, skinfold thickness and body fat percentage, are reported in school-based overweight prevention studies (Altenburg, In progress).^1^ To reduce heterogeneity between childhood overweight prevention intervention studies, it is important to develop a set of outcomes—known as a Core Outcome Set (COS)—that are relevant and meaningful to key stakeholders (called “actors” herein) including patients in clinical trials or participants in health promoting interventions, their caregivers and those involved in decision-making for both obesity prevention policy and practice (9, 10). Importantly, a COS is considered a list of fundamental outcomes, but not exhaustive: researchers can measure additional outcomes they believe are relevant to include in the evaluation of their intervention study (11). When studies include this minimum set of outcomes in their measurement and reporting, evidence synthesis from studies can be improved, contributing to evidence-based recommendations for policy and practice (e.g., policies around implementing healthy school lunches). Additionally, studies are more likely to measure outcomes appropriate to patients/participants, their caregivers, decision-makers and researchers (9).

To ensure a COS is relevant and meaningful to all key actors, including patients/participants, it is considered best practice to involve such actors in the development process of COS pertinent to them. COS developed for children's health conditions can vary in how children contribute meaningfully and fully to the COS development process. COS outcome papers from Bruce et al. (12) and Harman et al. (13) presented some of the early COS on children's health that include children's perspectives in the development process through visual and interactive methods (e.g., drawing and sharing through sticky notes). However, methods for inclusion are often not fully described to allow replication or to share practical strategies with others. Specifically, there is a lack of description for ways to involve children in the COS developments' ranking/rating stage. Article 12 of the United Nations Convention on the Rights of the Child (14) recognizes the value of every child having the right to express their views in matters affecting them. It is essential to develop and report on methods to allow children to contribute fully and meaningfully to a COS.

We developed a protocol for establishing a COS for primary/grade school-based intervention studies to prevent childhood overweight and obesity, including key steps that allow the input and perspectives of all key actors into the international consensus process (15). These actors include children of primary/grade school age, parents/caretakers, teachers and those working in the field of children's healthy lifestyle behaviors and childhood overweight prevention, such as healthcare professionals, policymakers and researchers worldwide (15). The present study obtained the perspectives of 8–13-year-old children—in various countries across the world—on outcomes that are relevant to them concerning school-based interventions that seek to improve children's healthy lifestyle behaviors, related to physical activity and diet. Methods are fully described to allow for replication and to inspire new ways to include children in a Delphi rating process.

## Methods

### Study design and participants

The Child Opinions on a Core Outcome Set for school-based intervention studies on stimulating healthy lifestyle behaviors (COCOS) is a qualitative study that includes focus group interviews, online or in-person, with children aged 8–13 years. Children at this age can generally understand the questions and are cognitively able to consider the topic under study and express their opinions at the level required for this study without the assistance of caregivers. Researchers in various countries were approached to contribute to the study, which resulted in data collection in the following six countries: the Netherlands, Scotland, Canada, South Africa, England, and the United States.

We aimed to sample at least 12 children in each of the six countries which provides a variety of regional and cultural backgrounds. Children were recruited using various strategies across countries. In the Netherlands, England, Scotland and South Africa, children were recruited through the researchers' network, i.e., through schools and families; in Canada, children were recruited through social media; and in the United States agricultural agents affiliated with the research institution and community leaders reached out to their network via listservs, word of mouth and strategically placed flyers. Interested children and their parents/caretakers received an information letter via email or in person. Informed consent from a parent/caretaker and each child was obtained in a variety of ways in line with each institution's ethics guidance. This included written or online through survey software consent or recorded verbal consent (children only). Ethical approval was obtained in all participating countries from the institute of which the primary researcher was affiliated: the Netherlands: Medical Ethics Committee of the VU Medical Center (no. 2020.071), Scotland: The University of Strathclyde School of Psychological Sciences ethics committee (72.27.04.2022.A), Canada: Children's Hospital of Eastern Ontario Research Ethics Board (REB Protocol No: 22/03X), South Africa: University of KwaZulu-Natal's Human Social Science Ethics Committee (HSSREC/00005476/2023), England: Edge Hill University's Science Research Ethics Committee (#ETH2223-0229), United States: Pennington Biomedical Research Center Institutional Review Board (FWA # 00006218).

### Procedures—interactive focus groups

Children participated in one standardized interactive focus group, in person or using a secure virtual platform, such as Microsoft Teams, Zoom, Google Meets. Focus groups were facilitated by two trained researchers and lasted ~1 h. Online focus groups were held with two to eight children per group, and in-person focus groups with six to eight children per group. Information on participating children's age and gender were collected at a group level and reported at a country level (Table 1).

**Table 1:** Study and participant characteristics.

	**Canada**	**England**	**Scotland**	**South Africa**	**The Netherlands**	**Unites States**
Sessions	6	4	5	6	6	3
Children per session (range)	32 (4–9)	27 (6–7)	14 (2–5)	48 (8)	23 (2–5)	15 (2–7)
Mean age, in years	10.6	9.4	10.3	10.1	10.3	10.5
Frequency age, in years	Unknown	8 = 17 9 = 14 10 = 17 11 = 17 12 = 12	8 = 2 9 = 1 10 = 4 11 = 1 12 = 4 (2 unknown)	9 = 17 10 = 17 11 = 6 12 = 8	8 = 3 9 = 6 10 = 2 11 = 8 12 = 3 13 = 1	8 = 0 9 = 5 10 = 2 11 = 4 12 = 4
Gender	31% girls, 59% boys, 10% unknown	50% girls, 50% boys	50% girls, 50% boys	63% girls, 27% boys	43% girls, 57% boys	40% girls, 60% boys

Supplementary material 1 includes the step-by-step protocol for the interactive focus groups. First, children were introduced to the study and topic using an animation video (link to English version: https://youtu.be/FtAN43MfS0E), developed using Vyond animation software and pilot-tested with Dutch children (15). This animation gave an overview of the study, what research is and who the researchers are, and explained intervention programmes aimed at improving healthy lifestyle behaviors by providing examples of different types of programmes (e.g., programmes that teach children about keeping their body and mind healthy, programmes that support the head of the school to offer more physical activity and physical education at school). The animation then explained that all sorts of information (i.e., “outcomes”) can be collected to determine if an intervention programme is successful which we articulated as “if it changes anything in the lives of children”. Then, using a vignette approach (16), children were given a hypothetical scenario involving the animated children on screen and were asked to brainstorm as a group ideas for a “healthy lifestyle programme” for the children in the animation video. Children wrote their ideas on a white board; for online focus groups methods like sticky notes on Google Jamboard were used and for in-person focus groups flip-over paper charts were used.

Additionally, the “superhero” exercise was conducted to check whether children understood the explanation of *outcomes*. Children were asked about their superhero: the superpower(s) it has, what the superhero would learn at superhero school and how we would see whether the superpower(s) have been improved at school. For example, children indicated that their superhero could jump very high, and that we could see improvements when they were able to jump even higher than before.

Second, children continued with the vignette and brainstormed outcomes they would consider important to measure for the children in the animation after they had participated in the “healthy lifestyle programme”. Subsequently, the children were asked to decide on the importance of each of the mentioned outcomes on a 9-point Likert scale using the traffic light system where red corresponds with a score of 1–3 (not important), orange with a score of 4–6 (important) and green with a score of 7–9 (very important), which is the approach previously used by Kirkham et al. (17, 18), Harman et al. (13), and Reilly et al. (19). The outcomes with a green (i.e., very important) rating were explained to the children as those they would like to know about themselves when participating in any of the mentioned intervention programmes in the video. For both the brainstorming and the importance scoring, children were asked to first think about this individually and subsequently share their ideas and opinions in the group. It was explained that there are no “wrong” answers regarding both outcomes and importance scoring, Children were encouraged to report the outcomes they themselves considered important. All ideas and opinions were summarized on either an online whiteboard (e.g., Google Jamboard) or in-person flipcharts.

### Data analysis and interpretation

The lead researchers combined the outcomes reported by children in the different focus groups, first per country, and subsequently across countries, merging all similar outcomes. The lists of merged outcomes per country and overall were reviewed and approved by the primary researcher(s) in each country. For each identified unique outcomes, we summarized the frequency of reporting and the overall importance score, both on a country level and across all countries. According to the definition of consensus from the Core Outcome Set-STAndards for Reporting guidelines-2 (17), we considered an outcome as “important” when ≥70% of the children scored the outcome as “very important” (green) and <15% of the children scored the outcome as “not important” (red). We considered an outcome as “not important” when ≥70% of the children scored the outcome as “not important” (red) and <15% scored the outcome as “very important” (green).

## Results

Table 1 shows the study and participant characteristics per country. In total, focus groups were conducted in 6 countries, including a total of 159 children: Canada: *n* = 32, England: *n* = 27, Scotland: *n* = 14, South Africa: *n* = 48, the Netherlands: *n* = 23, United States: *n* =15. In Canada, Scotland and the Netherlands, focus groups were held online and included two (the Netherlands, Scotland) to nine (Canada) children per focus group. In England, South Africa, and the United States, focus group sessions were held in-person, and included two (United States) to eight (South Africa) children. Children's average age ranged between 9.9 (England) and 10.6 (Canada) years and the percentage of participating girls ranged from 31 (Canada) to 63 (South Africa).

### Child-identified outcomes—per country

**Figure 1:**
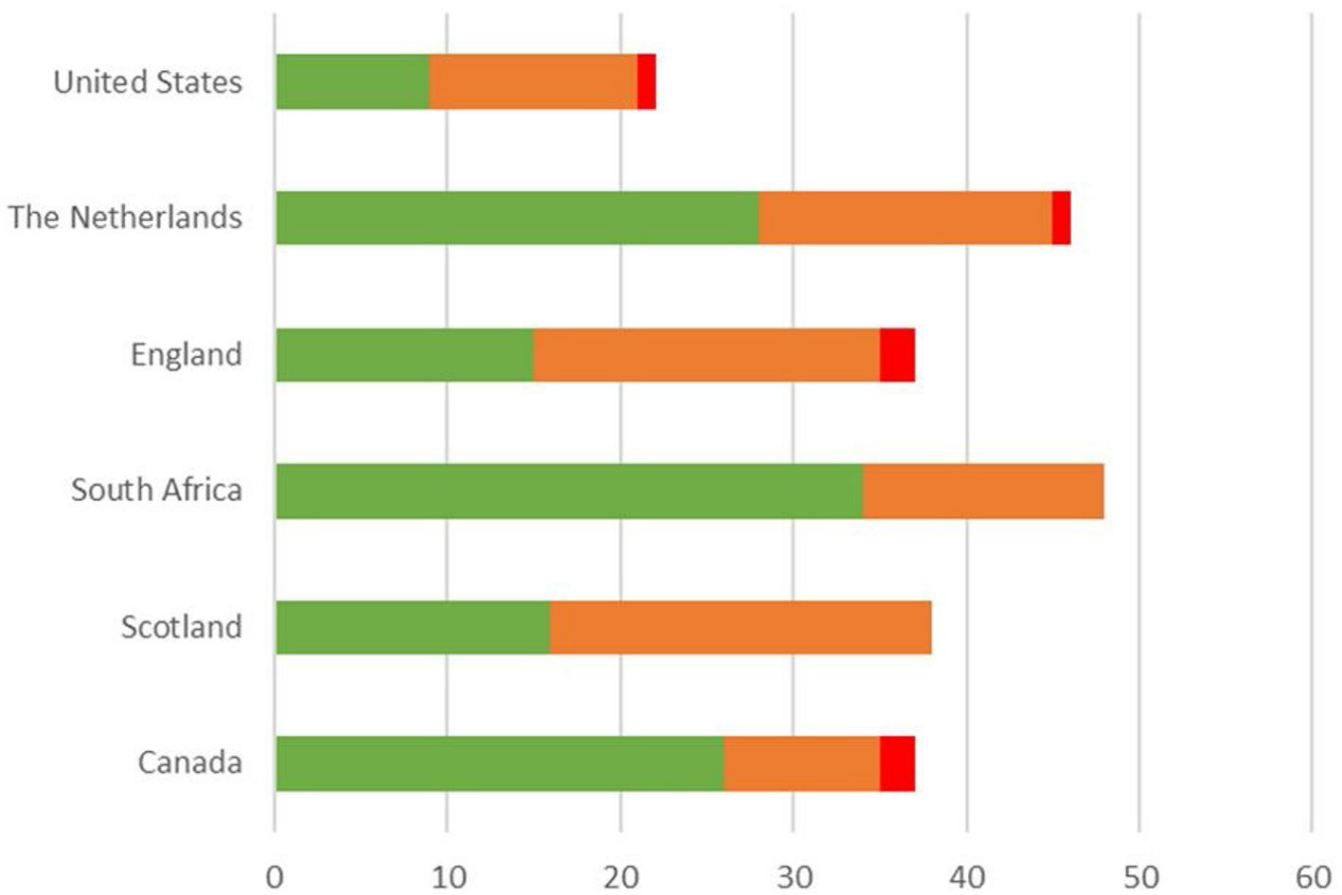
Total number of outcomes* reported by children in each of the participating country, after merging similar outcomes per country. *Numbers in green represent outcomes that were rated by children as “very important”, in orange as “important” and in red as “not important”.

Supplementary material 2 shows the list of merged outcomes per country and the importance ratings for each merged outcome, indicating the number of children that scored the outcome as “very important” (1), “important” (2) and “not important” (3), overall (per country) and per focus group. Figure 1 shows the total number of outcomes per country reported by children (grouped by rating, i.e., “very important”, “important” and “not important”), after merging similar outcomes per country. The number of outcomes reported by the children in each country ranged between 22 (United States) and 48 (South Africa). The number of outcomes that children overall (per country) scored as “very important” varied between nine (United States) and 29 (the Netherlands). The number of outcomes scored as “not important” varied between zero (Scotland, South Africa) and two (Canada, England).

The top-5 most frequently reported outcomes per country that children rated as “very important” were:

- Canada: being happy, being healthy, making children active, making children full of energy, to be more focused (reported in 3–4 out of 6 focus groups);
- England: be healthy, be more happier, strong lungs (only three outcomes reported in more than 1 focus group; i.e., reported in 2 out of 4 focus groups);
- Scotland: more happier, make you fitter, healthier (only three outcomes reported in more than 1 focus group; i.e., reported in 3–4 out of 5 focus groups);
- South Africa: ability to buy healthy food from tuckshop, more compulsory physical education (PE) and sports every week, help people eat better, drink more water in school, more fun activities like reading in the library (reported in 3–6 out of 6 focus groups);
- The Netherlands: fitness, being outside, having fun, body fat, making friends (reported in 3–4 out of 5 focus groups);
- United States: None of the outcomes scored as “very important” were reported in more than one focus group; reported outcomes consistently reported as “very important” by >5 children in one focus group included eating healthy foods, being better at sports, participating in more sports.

### Child identified outcomes—across countries

Supplementary material 3 shows the list of merged outcomes across the participating countries and the importance ratings of each merged outcome, indicating the number of children that scored the outcome as “very important” (1), “important” (2) and “not important” (3), overall (i.e., across countries) and per country.

**Figure 2:**
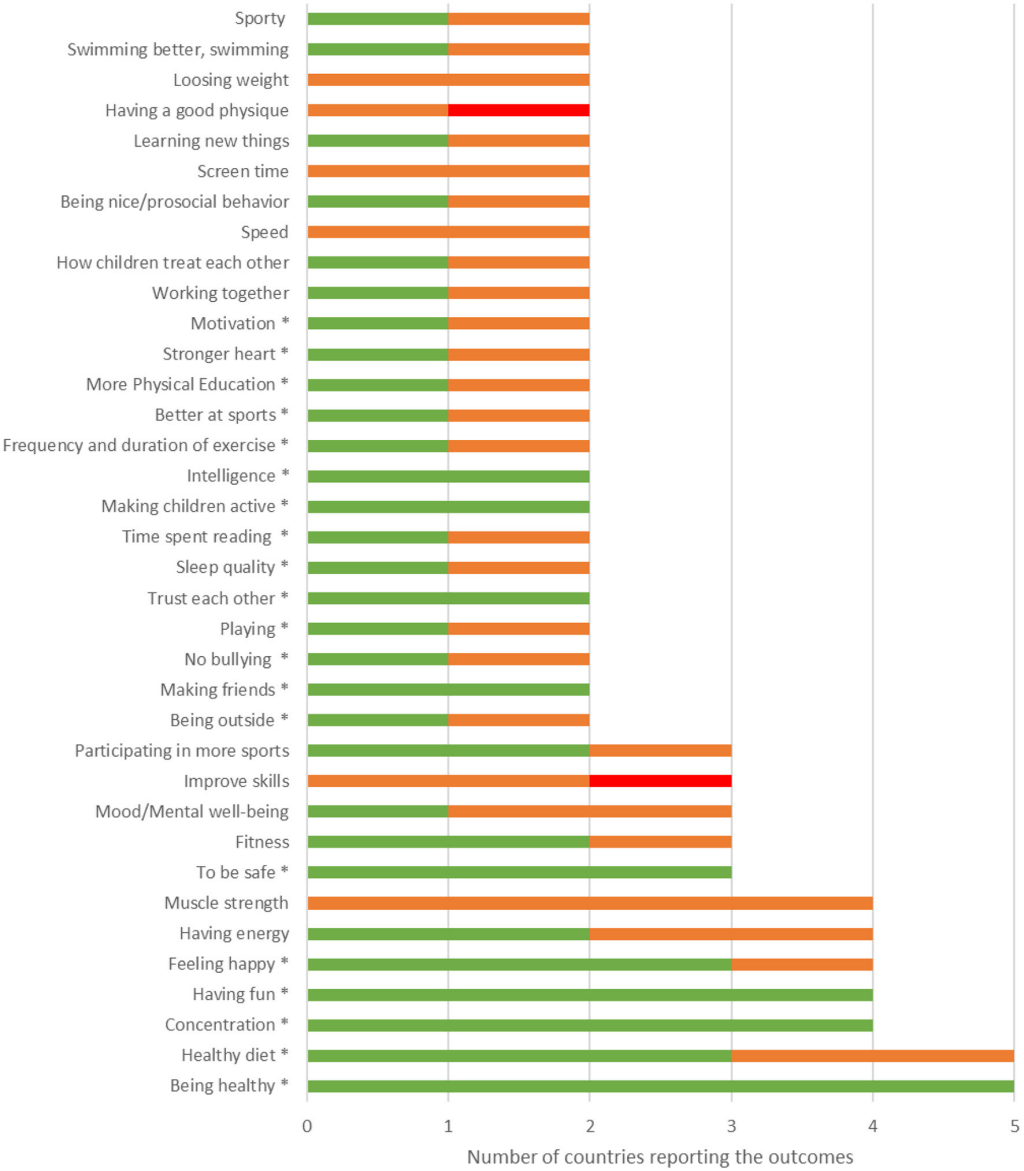
Number of countries reporting outcomes that were reported by children in at least two out of six participating countries, after merging similar outcomes per country, and importance ratings of counties that reported the outcome^a^. *Indicates outcome was overall rated as “very important”. ^a^Numbers in green represent outcomes that were rated by children as “very important”, in orange as “important” and in red as “not important”.

In total, children reported 170 different outcomes. Figure 2 presents the outcomes that were reported by children in at least two out of six countries (*n* = 36): two outcomes were reported by children in five countries, five outcomes by children in four countries, five outcomes were reported by children in three countries and 24 outcomes were reported by children in two countries. The majority of reported outcomes (*n* = 134) were only reported by children in one country.

Of the 36 outcomes reported by children in at least two countries, 20 outcomes received an overall rating of “very important”, of which nine outcomes were consistently rated as “very important” in all countries that reported the outcome (Table 2; see Figure 2 for importance ratings for all 36 outcomes). For example, the outcome “having fun” was reported in four countries and this outcome was rated as “very important” in each of these four countries. One outcome, i.e., “being healthy”, was reported in five countries and was rated as “very important” in all five countries that reported the outcome. Sixteen of the 36 outcomes received an overall rating of “important”, of which 14 outcomes were rated as “(very) important” in all countries that reported the outcome (see Figure 2). None of the outcomes provided by children in at least two countries received an overall rating of “not important”.

**Table 2:** Child-reported outcomes that were identified by children in at least two countries and overall rated as “very important”.

**Child-reported outcome**	**Frequency reported^a^**	**Overall importance rating^b^**
Being healthy	5	1(71), 2(4), 3(1)^*^
Healthy diet	5	1(74), 2(19), 3(4)
Concentration	4	1(33)^*^
Having fun	4	1(36), 2(3), 3(1)^*^
Feeling happy	4	1(50), 2(5), 3(1)
To be safe	3	1(14)^*^
Being outside	2	1(17), 2(1), 3(1)
Making friends	2	1(15)^*^
No bullying	2	1(4), 2(1)
Playing	2	1(6), 3(1)
Trust each other	2	1(6)^*^
Sleep quality	2	1(9), 2(1)
Time spent reading	2	1(20), 2(5), 3(2)
Making children active	2	1(20), 2(4), 3(1)^*^
Intelligence	2	1(10)^*^
Frequency and duration of exercise	2	1(10), 2(1)
Better at sports	2	1(9), 2(1)
More physical education	2	1(28), 2(6), 3(4)
Stronger heart	2	1(17), 2(1), 3(1)
Motivation	2	1(9), 2(3)

^a^Frequency reported indicates the number of countries in which the outcome was reported by children.

^b^Summary importance rating, summing the number of children (across countries) scoring the outcomes as “very important” (1), “important” (2) or “not important” (3) (according to the traffic light system). An outcome was considered as “important” when 70% of the children scored the outcomes as “very important” and <15% of the children scored the outcome as “not important”.

^*^Indicates consistency in the overall rating of “very important” across counties in which the outcome was reported by children.

## Discussion

Our study aimed to obtain the international perspectives of 8–13 year-old children on outcomes that are relevant to them in relation to school-based interventions that seek to improve children's healthy lifestyle behaviors, related to physical activity and diet. Our findings demonstrate that, after being explained about all relevant concepts such as interventions and outcomes, children are capable of reporting outcomes that they think are important to measure. Children reported a wide range of outcomes, not only related to physical and mental health, but also related to enjoyment and social skills, such as having fun and making friends.

Almost half of the top-20 of outcomes reported by children in at least two countries are mental, social or cognitive aspects of health and well-being, e.g., “having fun”, “feeling happy”, “concentration” and “making friends”. This is in contrast with the finding of our systematic review summarizing outcomes currently reported in studies that evaluate the effects of school-based interventions that aim to prevent childhood overweight and obesity, which demonstrate that the top-20 of most frequently reported outcomes does not include those related to children's mental health (see text footnote ^1^). None of the studies included in the systematic review reported outcomes that could be related to “having fun” or “making friends”. Child-reported top-20 outcomes related to children's physical health include “being healthy”, “muscle strength” and “fitness”, but not outcomes related to children's body weight or fat mass. Again in contrast, the top-20 of most reported outcomes in the extant literature up to 2024 relate to children's body weight or fat mass (e.g., BMI, weight status, body fat, waist circumference) (see text footnote ^1^). An important note to the comparison with our systematic review is that the review included studies evaluating school-based interventions to prevent overweight and obesity in children, whereas in the focus groups with children we reworded this as school-based interventions aimed at improving children's lifestyle behaviors, therefore not explicitly focusing on or mentioning overweight and obesity. Nevertheless, in some of the focus groups, children mentioned outcomes such as “losing weight” and “body weight”. Our findings suggest that those researching childhood overweight or obesity prevention may not measure or report outcomes that children consider to be relevant (i.e., socioemotional and cognitive well-being and health), whereas the outcomes they do include in evaluation studies are mostly overlooked or ranked with low importance by children. All outcomes reported and rated as “very important” by children will be included in a Delphi consensus process. Key actors including parents/caretakers, school staff, policymakers, and researchers will be invited to work toward consensus on an agreed set of core outcomes to be measured and reported in all future school-based intervention studies aimed at improving children's lifestyle behaviors and preventing childhood overweight and obesity (15). The Delphi process will reveal whether the outcomes considered “very important” by children are also very important to other actors, including academic researchers.

Although the top-5 most frequently reported outcomes per country that were rated as “very important” by children had some overlap, most of the top-5 outcomes were country-specific. Notably, the majority of outcomes were only reported by children in one country: about half of the outcomes reported by children in the Netherlands, Canada, Scotland and England were only reported in their own country (53, 51, 51, and 46%, respectively), whereas more than 2/3 of the outcomes reported by children in the United States (68%) and South Africa (79%) were only reported in their own country. Of the seven outcomes reported by children in at least four (out of six) countries, only two and three outcomes were reported by children in the United States and South Africa, respectively. In addition to country differences in frequency of reporting, of the 20 outcomes reported and considered as “very important” in at least two countries, only nine were consistently reported as “very important” by children in each country reporting these outcomes. The heterogeneity of outcomes and importance rating might be explained by differences in cultural values with respect to norms surrounding conceptions of what constitutes a state of “healthiness” and associated healthy lifestyle behaviors, ways of talking about health, and ways in which it is experienced by children. The country-specific difference in outcomes and importance ratings might suggest that children in the different countries have different priorities in terms of healthy lifestyle behaviors, which might call for a country-specific set of outcomes in addition to the international set of agreed core outcomes to be measured and reported in future school-based intervention studies aimed at improving children's lifestyle behaviors. However, it should be noted that children were not asked to reflect on outcomes mentioned by their peers in other focus groups and/or in other countries.

The methods applied in our focus groups worked very well in explaining complex concepts related to interventions and outcomes and obtaining valuable perspectives from children regarding outcomes they consider important to measure when evaluating interventions aimed at improving their healthy lifestyle behaviors. The animation video with a vignette approach helped children think about outcomes for primary school children in general, emphasizing that they do not need to relate this to themselves. Our findings demonstrate that children were able to discuss a wide range of different outcomes. The superhero exercise and using toys for the traffic light exercise also connected to the children's experiences and energized them throughout the focus group. However, the finding that children scored most of their reported outcomes as “very important” and almost none were rated as “not important” suggests that children may exhibit a positive bias toward their own opinions and those of their peers in their focus group. Although children scored the importance of each of the reported outcomes individually, as they could see and hear how the other children scored each outcome, they might have felt to pressure to score the outcomes as “(very) important” with the desire to have a positive impression on their peers or to conform.

A seminal COS development paper on a pediatric health condition included children at two stages of the COS development process. A protocol paper for COS development by Harman et al. (20) captured children's opinions using visual techniques, including asking the children to draw pictures and interact with apps on a tablet. The study team's subsequent papers conducted semi-structured interviews with children, a survey to rate outcomes using a traffic light system and online sticky notes (12, 13). Based on information from the Core Outcome Measures in Effectiveness Trials (COMET) imitative website (http://www.comet-initiative.org; July 2024), other methods for collecting opinions of children include one-on-one interviews, drawing treatment timelines, Technology of Participation method, using creative activities such as “drawings, stories and a tablet”, or having adolescents involved in the formal Delphi process. The successful involvement of children in the present and previous COS developmental studies (13, 20) can inform other “child health” COS initiatives.

Strengths of the current study include obtaining perspectives of children across six different countries worldwide and the application of a standardized and structured step-by-step protocol that could be implemented virtually or in-person. Through playful methods, such as an animation video including a vignette approach, a superhero exercise, and using toys for the traffic light rating, this study ensured that children could contribute in a playful way and to the best of their understanding. Finally, all steps in the data analysis and interpretation were checked by the primary researcher(s) in each country, ensuring that the data interpretation corresponds with the perspectives of the children in the different countries.

A key limitation in this work is the selection and reach of countries and sites. The sites were drawn from the COS developers” own research networks, no other criteria were applied. While we contacted other researchers in countries, including in Asia and Oceania, many who were interested were limited by lack of funding and/or human resource to run the focus groups. We note that it is common for COS development to be unfunded, under-funded and be run as a “passion project” of the developers. Nonetheless, those who can do that are in a privileged position, thus we advocate for research funding opportunities and institutional support to encourage these efforts, particularly for those in low-to-middle income countries, and our next steps in our COS development will aim to include those geographic areas underrepresented in the present COS development step. Some focus groups were done in person and others online. While there are strengths in both approaches, it is not clear whether the setting would mean a difference in what the children talked about and how. For example, children participating in online sessions may have been less influenced by their peers, and therefore give their own opinion, compared to in-person sessions, but at the same time less engaged in the session. However, feedback from facilitators did not report a lack of engagement. Facilitators reported the opposite, in fact, with children being easily and willingly able to participate through the variety of interactive methods used. Furthermore, recruitment of children was done in a variety of ways which also could affect how the children talk about health-related issues. Whether the children knew each other differed by each site and sometimes by focus group, which might have affected the extent to which children freely expressed their opinion. Additionally, our convenient recruitment strategy, mostly relying on the researchers” network and social media, may have resulted in selection bias. Another limitation is that we did not go back to the participating children to check whether we interpreted the reported outcomes correctly. Instead, the facilitating researchers at each site checked this. Finally, although children were encouraged to give their own opinion regarding outcomes and importance ratings, children may have rated outcomes more positive to please their peers.

## Conclusion

This study demonstrated that including children from multiple countries can contribute meaningfully and fully in the process of developing a COS when child-friendly methods are applied. Including children's voices provided relevant insights in which outcomes were considered relevant and important by children, including differences and similarities in outcomes and importance ratings across countries. The most frequently reported outcomes that were overall rated as “very important” by children were: “being healthy”, “healthy diet” (five countries), “concentration”, “having fun” and “feeling happy” (four countries). All outcomes reported by children in at least two countries and rated as “very important” should be considered in a COS for school-based intervention studies that aim to improve children's healthy lifestyle behaviors and prevent childhood overweight and obesity.

## Data Availability

The original contributions presented in the study are included in the article/Supplementary material, further inquiries can be directed to the corresponding author.

## Footnotes

^1^Altenburg TM, De Vries LW, Grooten IJ, Van t Hooft J, Van Deutekom AW, Staffleu A, et al. Outcomes reported in school-based intervention studies on preventing childhood overweight and obesity: a scoping review. In progress.
