# Supplementary material for: Child opinions related to a core outcome set for school-based healthy lifestyle behavior interventions: the COCOS study
Source: Front Public Health. 2025 Apr 16;13:1519467. doi: 10.3389/fpubh.2025.1519467 (PMC12040649; doi:10.3389/fpubh.2025.1519467)
Supplement: Supplementary file 1 [file Table_1.docx]

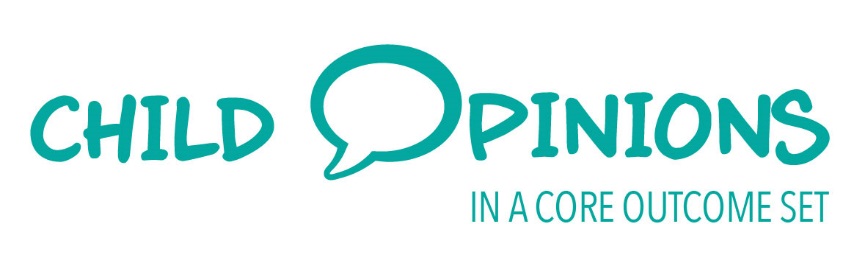


**WORKING PROTOCOL COCOS – ONLINE FOCUS GROUPS**

# Aim of the focus group meeting

The aim of the focus group meeting is to obtain children’s opinions on which outcomes they think are important when evaluating a school-based studies aimed at improving children’s healthy behaviours.

# Target population

Children will be 9-12-year-olds (attending primary school; no exclusion criteria regarding weight status). Preferably focus groups will be organized with children from different areas/backgrounds: e.g. children from lower and higher SES areas, children from cities and smaller villages.

# Necessities

- Two facilitating researchers
- Small incentives for the children
- Time keeping device (e.g. phone)
- Three items for each child in colours: green, yellow and red
- Online facilitation:
  - Zoom meeting
  - Online white board such as Google Jamboard (jamboard.google.com)
    - Check the access rights of the Jamboard: everyone with the link should be able to make changes to the Jamboard. Note that a Gmail account is needed to create and share a Jamboard.
    - Templates for the pages are at the end of this document
- In-person facilitation
  - Flip-over with multiple sheets
  - Content of different sheets are similar to the Jamboard templates

# Procedures

Following explanation of the study, children will be asked which outcomes they consider relevant in school-based studies aimed at improving children’s healthy behaviours, using an animation video and additional questions. Next, the children will rate the importance of all mentioned outcomes, using a traffic light system. In both steps, children will think about this topic individually and subsequently they will share their ideas and opinions in the group. Consensus on the complete list of outcomes as well as the importance of all outcomes will be reached through discussion.

# Preparation

- Share the information letters and animation video with the parents and children.
- Collect the informed consent forms from the parents and children, confirm if all the children who are present have a complete consent form.
- In case of online focus group: Open the Google Jamboard. In case of in-person focus group: collect the prepared flip-over sheets.
- In case of online focus group: Start to prepare the online Zoom environment and check if everything is working (with help of the parents) 10 – 15 minutes before the meeting.
- Roles and responsibilities: One of the researchers (researcher 1) is the main facilitator and is responsible for collecting the child-identified outcomes, importance rates and children’s explanations (keep notes on the laptop). The other researchers (researcher 2) is responsible for keeping the time, to warn the other researcher (researcher 1) when there are 2 minutes left of the maximum time for each round. Additionally, researcher 2 assists in facilitating the focus group.
- Optional for online meeting: Ask the children/parents before the meeting if they have three toys/items in the colours green, orange (or yellow) and red. It is preferred to ask this during the meeting, (i.e. before the importance ranking phase) as searching for the three items will be a nice game-like variation of activities without having to take an actual break.

# During the meeting

*Note: the working protocol is outlined for an online focus group; for an in-person focus group, a flip-over is used, with multiple sheets.*

### Introduction (max. 20 min.)

- Researchers introduce themselves, and ask children to introduce themselves: Each child states their name and age, we only collect the age and gender on group level (not connected to the name). You can additionally ask the children to share a hobby, to make it a bit more personal, the researchers can also share a hobby.
- Explain the intention of today’s meeting and emphasize that there are no right or wrong answers, the children may say what they think and cannot make mistakes.
- Play the animation video.
  - *In a ZOOM-meeting: Share screen > advanced > video > Select file*.
- We go to Google Jamboard, by sharing the link in the chat and asking the children (with help from their parents if needed) to click it and open the first Jamboard page (‘introduction page’); If possible ask children to split their screen with on the one side the Jamboard and on the other side the Zoom screen. If this is not possible, it’s ok to move the Zoom screen to the background.
- To check if everybody has access and rights to the Jamboard and to make everyone familiar with the software program we first ask the children to type their name in a sticky note on the first page.
- When every child knows how to post a sticky-note, move on to the second Jamboard page, which is the ‘intervention & outcome’ page.
- Check if children understand what is explained in the video by asking further questions. Make sure these questions are directed towards the group of children in the animation video, by asking children to answer the questions on behalf of the kids in the video rather than themselves in the first instance. This will prevent children feeling embarrassed to talk about outcomes they might consider important for themselves.
  - First, check if children understand what an intervention or a ‘healthy lifestyle programme’ is (“*What is your understanding of an intervention?”*), and check if they understand what an ‘outcome’ is (“*What is your understanding of an outcome?”*).
    - You could ask the – in preparation of this meeting – if there was recently an intervention or program at their school. Subsequently, you might use the same terms and further explain what an intervention is by talking about children’s own experiences at school.
    - You could use the example of a ‘superhero training/intervention’. Ask the children about the qualities of a superhero (e.g. he/she can fly, jump high, see through walls). Subsequently ask how a superhero could be trained to (further) develop his/her superhero qualities (e.g. flying/jumping lessons). Finally, ask what should be measured in order to know whether the training/intervention worked, whether the superhero succeeded at learning these qualities (e.g. measure how long the superhero can fly or how far the superhero can jump).
  - Move on to the third Jamboard page (‘intervention; page)
  - Second, ask the children to help designing an intervention program for the children in the video, this will help them to think about an intervention relevant to them (and later on about outcomes relevant to them): *“If you had the change to design an intervention at school, what would it look like?”*
    - Let the children think about this individually and let them put their ideas on sticky notes in the Jamboard.
    - When all children have put their ideas on sticky notes, discuss these ideas with the whole group.

### Brainstorm phase (max. 10 min)

- Ask the children what would be important to know about the animation video-children, to know whether the program worked (*“Which outcomes are important for the children in the video, who participated in any of the mentioned intervention programs?”*).
  - Let the children think about this individually and let them note their ideas on a paper – not yet on a sticky not in the Jamboard (to stimulate independent brainstorming by all children).
- When this is understood and goes well, you may additionally ask the children to think about themselves, to get more ideas of relevant outcomes (“*What would you like to know about yourself when you participated in an intervention program at your school? What information about your health, your health behaviors and your life is important to you?”*)

### Idea-sharing phase (max. 15 min.)

- Move to the fourth Jamboard page, which is the ‘outcomes’ page, part I.
- The children share the outcomes one-by-one, i.e. each child shares one outcome that has not been mentioned before and then the next child shares one outcome that has not been mentioned before etc. In this way, the children might stimulate each other to think of other/new outcomes.
- The children can type the outcome on a sticky-note on the Jamboard, the researcher can assist.
- The facilitating researcher (researcher 1) will keep track of the outcomes/concepts that are put forward and make sure that all outcomes are clear and not overlapping with another outcome. If necessary, ask clarifying questions.
- This phase ends when no new outcomes are mentioned.

### Importance ranking phase (max. 15 min.)

- During this phase the children will decide for themselves what the most important outcomes are. Ask the children to go and find three items or toys in their house, small enough to show on screen: one red, one orange and one green item/toy. These items/toys represent the traffic light.
- Put the Jamboard traffic light coloured boxes in the middle of the Jamboard ‘outcomes’ page (boxes are visible in the corners of the Jamboard page (part I)), and delete the blue box (see fourth page, part II).
- Move all outcomes (on sticky notes) to the right side of the Jamboard page (in the red box).
- One-by-one, read all outcomes and ask the children how important they consider each specific outcome. Ask children to indicate this by showing the toys/items in the colour of the corresponding box:
  - green box reflecting a ‘very important’ outcome
  - orange box reflecting a ‘neutral’ outcome
  - red box reflecting an outcome that is ‘not important’
- If consensus is reached: move the outcome to the box that the children agree on.
- In case of disagreements: facilitate a discussion to obtain consensus on whether an outcome is important or not.
  - It is especially important to obtain consensus regarding the outcomes that are considered ‘very important’ by (one of) the children.
  - If consensus cannot be obtained, make a note including children’s different opinions on whether or not children think the outcome is (not) important.

*Note to researchers:* The colours in the traffic light correspond to ratings on a 9-point Likert scale that will be used in the Delphi study among adult stakeholders: Red corresponds with the ratings 1 to 3 (not important), orange corresponds with ratings 4 to 6 (important) and green with ratings 7 to 9 (very important).

### Closing the meeting (5 min.)

- The researcher thanks the children and the children get a small incentive for their cooperation.

# After the meeting

- Make sure all notes, explanations and the Jamboard pages are saved, copied and locked.
- All the personal information is saved properly with regards of privacy (we only collect gender and age on a group level)

## Google Jamboard pages

#### Page 1- Introduction page


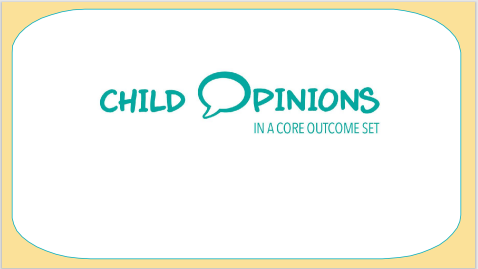


During the familiarization with the program everybody can post a sticky note on this page with their own name.

####
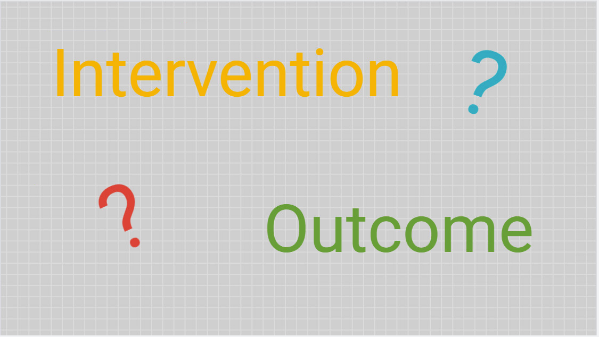
Page 2 – Intervention & Outcome page

During the introduction phase, children will be familiarized with (types of) interventions and outcomes.


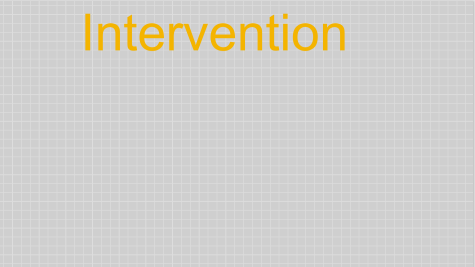


#### Page 3 – Outcomes page

The children can put their ideas regarding their own intervention (program they think is relevant for them/the children in the video), they can post sticky notes with what the intervention would consist of on this page.

#### Page 4 – Outcomes page, part I


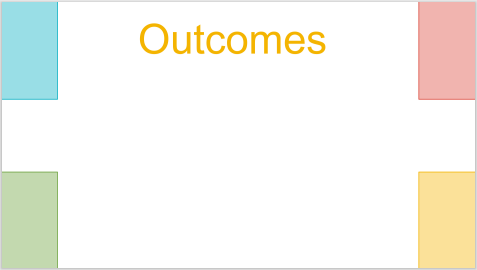


In this page the children post (on a sticky note) all possible outcomes they can think of.

####
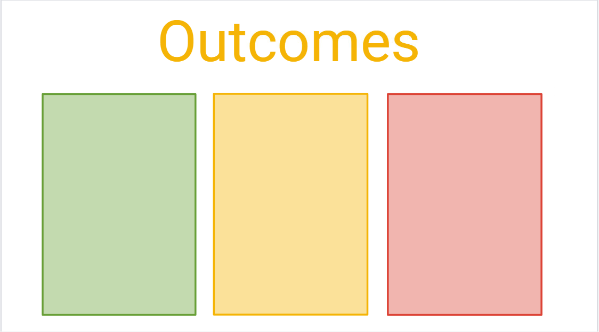
Page 4 – Outcomes, part II

This is the same age as above (because the sticky notes remain on this page), but with the traffic light coloured blocks in the middle of the screen (blue box deleted). All sticky notes are moved to the right side (in the red box). The researcher moves the outcomes (on sticky notes) the to the box that the children agree upon.
